# Optimizing the pharmacokinetics of sonidegib in small bowel syndrome and advanced basal cell carcinoma: Our solution

**DOI:** 10.1016/j.jdcr.2023.06.016

**Published:** 2023-06-20

**Authors:** Hailey Grubbs, Marianne Cortes, John Strasswimmer

**Affiliations:** a Dermatology Department, Broward Health Medical Center, Ft Lauderdale, Florida; b Nova Southeastern University Kiran C Patel College of Osteopathic Medicine, Davie, Florida; c College of Medicine, Florida Atlantic University, Boca Raton, Florida; d Strasswimmer Mohs Surgery, Delray Beach, Florida

**Keywords:** advanced basal cell carcinoma, alternate dosing, comorbidities, cutaneous oncology, dosing strategies, dysphagia, Hedgehog pathway inhibitor, intestinal malabsorption, pharmacokinetics, skin cancer, sonidegib, treatment, vismodegib

## Introduction

The Hedgehog pathway inhibitors (HHIs) vismodegib and sonidegib are indicated to treat recurrent locally advanced basal cell carcinoma (BCC) following surgery or radiation, or patients ineligible for surgery or radiation.^1^, ^2^, ^3^, ^4^ Recommended dosing is 1 capsule by mouth daily until significant toxicity or disease progression occurs.^1^^,^^2^ Vismodegib can be taken with or without food, but sonidegib is taken on an empty stomach, as high-fat foods significantly increase its absorption.^1^^,^^2^ This pharmacologic property may provide opportunities for alternate sonidegib dosing in patients with comorbidities affecting drug administration or absorption. We present a case where novel sonidegib dosing produced positive patient outcomes.

## Case report

**Fig 1:**
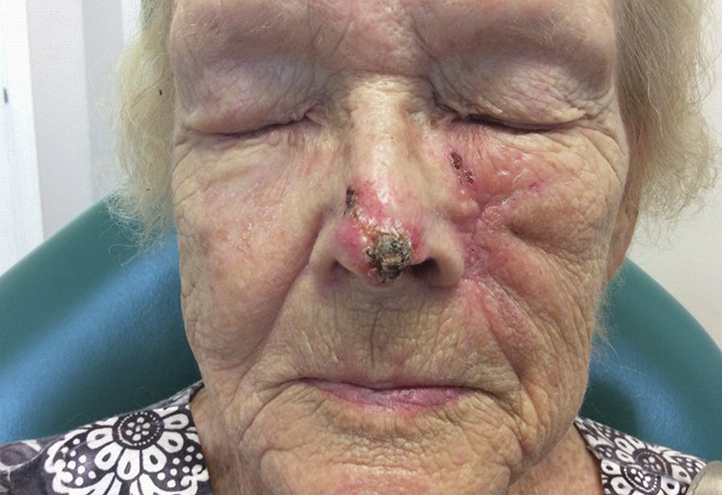
Infiltrative basal cell carcinoma in our patient prior to initiation of the sonidegib–ice cream combination. On the nasal tip, right nasal side wall, and left alar rim is a pink, pearly plaque with hemorrhagic crusting. Biopsy of this lesion revealed an infiltrative basal cell carcinoma. The smaller lesion on the left cheek represents an unrelated, biopsy-confirmed squamous cell carcinoma that was ultimately treated with Mohs micrographic surgery.

An 83-year-old woman with history of multiple keratinocyte carcinomas, dysphagia, and short bowel syndrome presented to our office with an eroded, ill-defined plaque on her nasal tip measuring approximately 2.0 × 1.3 cm (Fig 1). Biopsy revealed a recurrent infiltrative BCC. Prior therapies to this area included Mohs micrographic surgery, radiation, and salvage radiation. The patient’s short bowel syndrome and dysphagia precluded HHI administration as directed. Therefore, following an extended discussion of management options with consideration of HHI pharmacokinetics, we prescribed low-dose sonidegib combined with a fatty meal. Half a capsule (100 mg) of sonidegib was added to 2 tablespoons of vanilla ice cream (12 g fat/118 g serving size) and taken once daily. Due to teratogenicity risk with sonidegib, preparation was performed by a family member of no reproductive potential using medical gloves and a razor blade.^1^ Tumor response and signs and symptoms of toxicity were monitored. Electrolytes, liver function, and creatine phosphokinase (CK) were evaluated 2 weeks before treatment initiation; at weeks 2, 4, and 8; and then monthly during treatment. The patient's informed consent for publication was obtained by the authors, and the work described was carried out in accordance with the Declaration of Helsinki.

**Fig 2:**
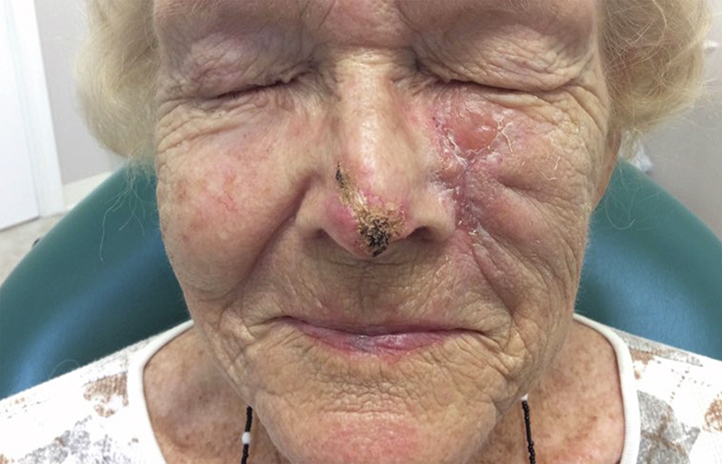
Infiltrative basal cell carcinoma in our patient 2.5 weeks after starting treatment with the sonidegib–ice cream combination. By 2.5 weeks of treatment, clinical improvement was observed in the size and amount of hemorrhagic crusting associated with the nasal basal cell carcinoma. On the left cheek, the unrelated squamous cell carcinoma is visible.

The patient’s positive clinical progress halfway through the first 5-week sonidegib treatment course is depicted in Fig 2.

**Fig 3:**
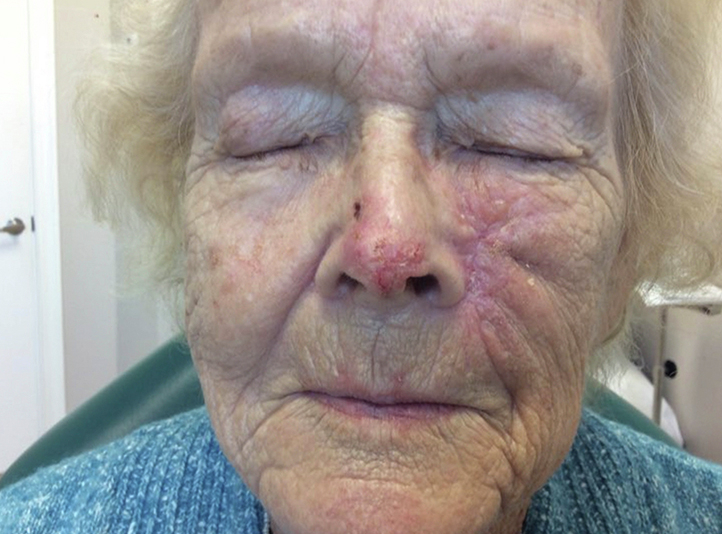
Infiltrative basal cell carcinoma in our patient 5 weeks after starting treatment with the sonidegib–ice cream combination. After 5 weeks of treatment, the patient experienced improvements in the size of the erythematous plaque and hemorrhagic crusting on the nose, and reported decreased pain in the area. On the left cheek is a healing surgical scar from the recent treatment of the unrelated squamous cell carcinoma.

By 5 weeks after treatment initiation, tumor size had decreased to approximately 1.2 × 0.8 cm (Fig 3). Around this time, the patient began experiencing nausea and muscle cramping. Her CK levels remained normal. A drug holiday was instituted with subsequent symptom resolution. After 5 weeks off therapy, tumor regrowth was noted, and sonidegib was restarted for another 5 weeks. In total, the patient completed three 5-week courses of sonidegib with 5-week medication holidays between each course. Bloodwork values remained within normal limits for the duration of treatment. After her third treatment course, the patient and her family were satisfied with her results and treatment was ceased. Over 1 year later, she denies further issues in the area.

## Discussion

Hedgehog signaling has been implicated in a number of cellular processes including cell proliferation and differentiation,^5^ and abnormal Hedgehog signaling is found in 95% of sporadic BCCs.^4^ HHIs, including vismodegib and sonidegib, work to treat locally advanced BCC by binding to Smoothened and preventing downstream transcription of glioma-associated oncogene.^1^, ^2^, ^3^, ^4^ Sonidegib and vismodegib are dosed as 1 oral capsule daily, swallowed intact.^1^^,^^2^ Due to the fat-soluble nature of sonidegib, we elected to use this HHI with ice cream, a high-fat food, to increase drug absorption and bioavailability in our patient with small bowel syndrome.^1^^,^^2^ Combining this with an easy-to-swallow food also allowed us to overcome her dysphagia. Knowing this combination would significantly increase drug absorption, we prescribed half a capsule to minimize toxicity risk. Using this regimen, the patient’s treatment goals were achieved.

The length of HHI treatment is determined by efficacy and tolerability and is patient-dependent. Some reports have demonstrated sustained response with treatment courses as short as 2 weeks,^6^ while others report tolerability and efficacy for over 4 years.^7^ Common HHI adverse events (AEs) include muscle spasms, alopecia, dysgeusia, fatigue, weight loss, and elevated CK, and, in the case of sonidegib, AE incidence may increase with concurrent food administration.^1^^,^^4^^,^^8^^,^^9^ Monitoring of electrolytes, kidney function, and CK during sonidegib treatment is recommended.^1^^,^^8^ Our patient required several drug holidays due to the occurrence of nausea and muscle cramping during treatment, but her CK values remained normal. In our clinical experience, the onset of sonidegib therapeutic response often precedes AE onset. Therefore, medication cycling in this way may improve tolerability. Using topical HHI formulations or multidrug HHI regimens may also increase efficacy and decrease toxicity in treated patients.^5^^,^^10^

Overall, our alternative treatment regimen was effective and acceptably tolerated in our patient with impaired gastrointestinal absorption and dysphagia. Further studies would be helpful in developing alternate sonidegib dosing schedules to minimize AEs, and in establishing dosing recommendations for sonidegib combined with high-fat foods to optimize bioavailability and minimize toxicity in patients with intestinal malabsorption.^8^ Implementation of personalized dosing regimens in the treatment of advanced BCC may be promising for patients with unique presentations that limit the feasibility of conventional treatment protocols.

## Conflicts of interest

None disclosed.
